# Task shifting of intravitreal injections from physicians to nurses: a qualitative study

**DOI:** 10.1186/s12913-021-07203-8

**Published:** 2021-10-30

**Authors:** Stine Bolme, Dordi Austeng, Kari Hanne Gjeilo

**Affiliations:** 1 Department of Ophthalmology, St. Olavs Hospital, Trondheim University Hospital, Trondheim, Norway; 2 Department of Neuromedicine and Movement Science, Norwegian University of Science and Technology (NTNU), N-7489 Trondheim, Norway; 3 Department of Public Health and Nursing, Faculty of Medicine, NTNU, Trondheim, Norway; 4 Clinic of Cardiology, St. Olavs Hospital, Trondheim University Hospital, Trondheim, Norway; 5 Department of Cardiothoracic Surgery, St. Olavs Hospital, Trondheim University Hospital, Trondheim, Norway

**Keywords:** Task shift, Intravitreal injections, Interview, Nurse, Qualitative

## Abstract

**Background:**

Intravitreal injections of anti-vascular endothelial growth factor are high-volume procedures and represent a considerable workload on ophthalmology departments. Several departments have tried to meet this increase by shifting the task to nurses. To maintain high-quality patient care, we developed a training program for nurses that certifies them to administer injections. This qualitative study aimed to evaluate whether the nurses were confident and in control after participating in the training program and whether they were satisfied with the training and the new task.

**Methods:**

Between 2014 and 2018, 12 registered nurses were trained in a tertiary hospital in central Norway. All the nurses were interviewed, either individually (*n* = 7) or in a group (*n* = 5). We analysed the interviews using Graneheim and Lundman’s qualitative content analysis.

**Results:**

Eight subthemes were clustered within four main themes: 1) procedure and challenges, 2) motivation, 3) cooperation and confidence, and 4) evaluation. The nurses felt confident and in control when administering injections but experienced moments of insecurity. The new task gave the nurses a sense of achievement, and they highlighted improvement of patients’ lives as positive. A greater level of responsibility gave the nurses pride in their profession. They had suggestions that could improve training efficiency but were overall satisfied with the training program.

**Conclusions:**

Our study showed that the nurses were satisfied with the training and that learning a new task led to higher self-esteem and increased respect from patients and colleagues. Suggestions to improve the training were identified; these should be considered before implementation by other departments.

## Introduction

Intravitreal injections (IVI) with anti-vascular endothelial growth factor (anti-VEGF) are an efficient treatment for several retinal diseases [1], and the use of anti-VEGF has had an exponential growth over the last two decades [2]. The treatment has not only had a major impact on eye health, it has also changed the division of labour in ophthalmology departments, as many have shifted the task over to nurses [3]. The basis for this is that a vertically staged task shift, with tasks transferred from a higher level of competence to a lower one, is a way to better utilize resources [4, 5]. The role of nurses is evolving [6, 7], and advanced nursing practice has enabled task shifting from physicians to nurses [8, 9]. However, it is still not common for nurses to perform surgical procedures independently. Therefore, nurses who administer intravitreal injections expand the role of nursing into a new area.

In 2019, with the first randomized controlled study, we were able to show that nurse-administered injections are just as safe and have the same positive effect as injections given by physicians [10]. Based on these results, we established a nurse-driven injection clinic at the University Hospital in Trondheim, Norway.

A successful nurse-driven injection clinic relies on satisfied nurses, as increased well-being raises the quality of the work [11] and leads to satisfied patients [12–14].

Job satisfaction among nurses is a recurring theme in the literature [15–17], but studies of nurses’ satisfaction concerning task shifting and training are scarce. To identify the thoughts and experiences of nurses certified to administer IVIs, we conducted a qualitative study. The aim was to explore their level of confidence and control after completing the training program and their satisfaction with the new task.

## Material and methods

### Design and sample

**Fig. 1:**
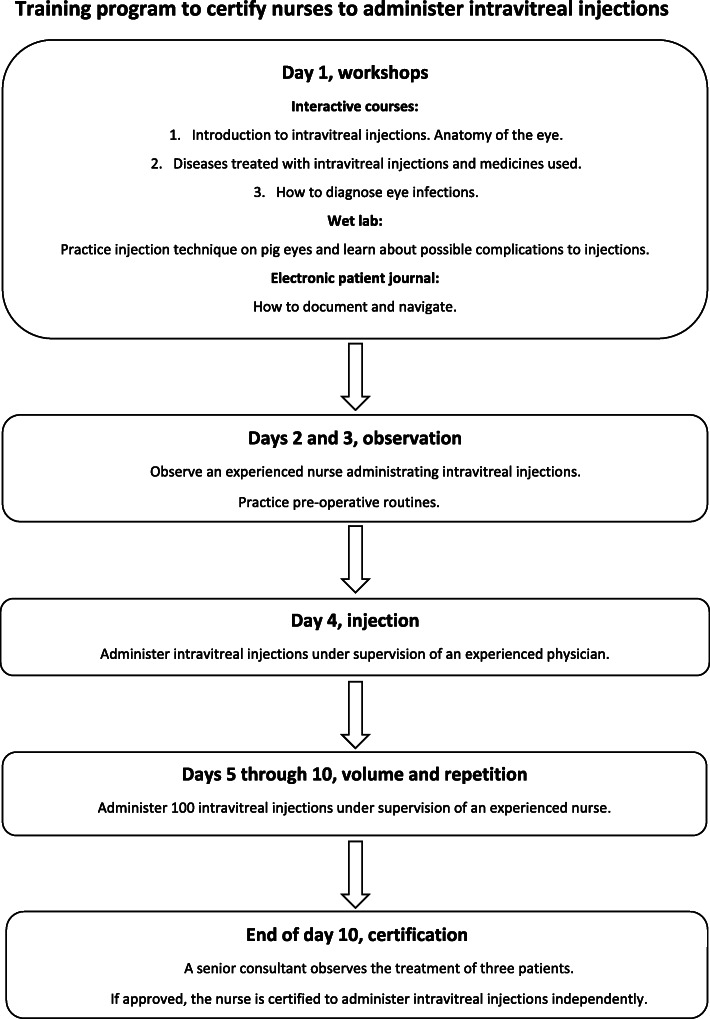
Training program to certify nurses to administer intravitreal injections. The final version of the 10-day training program

This qualitative study had an inductive descriptive design, with semi-structured interviews conducted individually and in a focus group. The study took place at a tertiary hospital covering about 750,000 inhabitants in Central Norway. We developed a training program in our ophthalmology department to certify nurses to administer IVIs independently. The final version of the training program lasts 10 days and comprises workshops, wet lab, and observation before nurses perform injections (Fig. 1).

From April through August 2016, seven out of a total of 12 nurses were interviewed individually. The idea was to use a total sampling strategy, and initially, only seven nurses were trained to administer injections. Later, five more nurses were trained, and these nurses were interviewed in a focus group in March 2019. Different interview approaches were chosen to enrich the data [18, 19]. The interviews lasted between 14 and 50 min. The characteristics of the participants and type of interview are given in Table 1.

**Table 1:** Characteristics of the participants

ID	Age at time of interview	Educational information	Years of ophthalmological practice at time of interview	Type of Interview
N1	31–40	Master	> 6	Individual
N2	21–30	Master	1–3	Individual
N3	31–40	Master	4–6	Individual
N4	> 40	Master	> 6	Individual
N5	21–30	Bachelor	1–3	Individual
N6	21–30	Master	4–6	Individual
N7	> 40	Master	> 6	Individual
N8	> 40	Bachelor	4–6	Focus group
N9	> 40	Bachelor	1–3	Focus group
N10	31–40	Bachelor	1–3	Focus group
N11	21–30	Bachelor	4–6	Focus group
N12	31–40	Bachelor	4–6	Focus group

The nurses were approached and asked face-to-face to participate by the first author, and no one declined.

### Data collection and analysis

The interviewer was the first author (SB), a female PhD student and part-time resident at the Department of Ophthalmology with no former interview experience. She attended a PhD course in qualitative research while performing the interviews and was trained and supervised by the last author, an experienced researcher in qualitative method. The interviews were carried out in the ophthalmology department. A semi-structured interview guide was developed prior to the individual interviews based on the research questions, previous knowledge, and literature on the task-shift concept. The interview guide was piloted in the interview with the first nurse and later used without revision. Before the focus group interview was conducted, the interview guide was further developed, based on the experience from the individual interviews and to adjust to the anticipated group dynamic. This resulted in fewer and more open questions. In the focus group, the first author was the moderator and the last author (KHG), who had never met the participants before, was an observer. The interviews were taped using a voice recorder and transcribed verbatim by the first author. Field notes on contextual information, thoughts, mood, and facial expressions during the interview were taken by the first author. Participants were not given the opportunity to give feedback on the transcripts or findings.

Both the individual interviews and the focus group interview were analysed using qualitative content analysis with an inductive approach, according to Graneheim and Lundman [20].

The first author read all the raw data several times to gain an overview. Then, the process was as follows: (1) the first step of coding was finding **units of meaning**; (2) the units of meaning were then **condensed** into fewer words; (3) the condensed units of meaning were clustered into preliminary **code groups**; (4) related codes were ordered into broader, higher-ordered **subthemes**; and (5) subthemes with similar meanings were grouped together at the highest level, called ‘**themes**,’ which the first and last author discussed and agreed on. The consistency of major themes was ensured by comparing data from the individual interviews and focus group interview. The analysing process resulted in a coding three, illustrated in Table 2 which gives an example of the units of analyses based on one of the branches. The second author (DA), a consultant ophthalmologist, contributed by confirming the final analysis and discussing which parts of the interviews would be highlighted. The analysing process was performed with two different tools. First, in 2019, the analysis was done with paper and marker pen. It was repeated a year later, after a course in qualitative research about the analysing process, using the data program NVivo 12, a computer-assisted qualitative data analysis software (QRS International). This approach was chosen to check whether the second analysing method gave the same results and to achieve rigor [21]. During the process, there was continual discussion between the first and last author, which brought valuable perspectives as the last author is an experienced qualitative researcher. The amount of data was considered saturated as the interviews had sufficient information power [22]. The consolidated criteria for reporting qualitative research (COREQ), a 32-item checklist for interviews and focus groups, was adhered to in the reporting of this study [23].

**Table 2:** Examples of the analysis

Meaning units	Condensed meaning units	Code groups	Subthemes	Theme
I feel like I do a better job when I’m working at the injection clinic than when I work in the outpatient clinic. I feel I make a difference when I administrate injections.	Really achieving something good when working at the injection clinic	Achieve something good	To make a difference	Motivation
When I’m in the injection room, I’m more aware of my blood sugar. In the outpatient clinic, we measure visual acuity and eye pressure and I can feel my belly rumbling, but it’s okay, we keep going. But if I’m in the injection room, I’m more aware of that it affects me negatively and that I have to eat something before I continue.	Have to be at the top of your game	Capability	Responsibility and respect	Motivation
I find that we are given a greater confidence in relation to a so-called physician assignment being given to us nurses, which I like very much, because it shows that people also have faith in nurses.	Given greater confidence from the department	Increased respect	Responsibility and respect	Motivation

## Results

The 12 participants, one male, had a wide age span ranging from 26 to 60 years. Their educational background was evenly distributed at bachelor’s and master’s levels. The nurses’ experience from clinical practice in ophthalmology ranged from two to 28 years, with a mean of eight years.

Eight subthemes emerged from the data, clustered in four main themes: 1) procedure and challenges, 2) motivation, 3) cooperation and confidence, and 4) evaluation. The themes and the eight subthemes are illustrated with quotations in the text and shown in Table 3.

**Table 3:** Description of themes and subthemes identified through the interviews

Main themes	Procedure and challenges	Motivation	Cooperation and confidence	Evaluation
Subthemes	Routine versus variation	Responsibility and respect	Working as a team	Continual learning
	Challenges	To make a difference	Confidence	Adjustments

### Theme 1: procedure and challenges

The nurses stated they felt confident at different stages in their training, and the ones with the most experience soon saw the injections as a routine instead of a challenge. Regardless of previous experience, the nurses had moments of insecurity.

#### Routine versus variation

The nurses expressed that learning a new task gave them increased variety.

“You get a larger repertoire and the work gets more varied.” (N7).

The group of nurses disagreed on this question, and the ones with the most experience said that the new task quickly became a routine. One nurse had hoped for a greater challenge and was disappointed.

“Every patient can be a challenge in themselves, but I will not claim to have large challenges in the injection room. It’s more of a routine.” (N1).

#### Challenges

Assessing whether patients had an eyelid infection was mentioned as a common source of insecurity. It was especially challenging if the patient came directly from a physician examination reporting that eyelid infection was not present while the nurse was convinced of the opposite.

“A patient came from examination and the journal note written by the physician said that there were traces of blepharitis, but that it was okay to inject. This leaves us nurses a bit insecure. If it is blepharitis we should not inject, and this is what we have been trained to think. And at the end, it is us who injects the needle into the eye. Of course, it is the physician’s responsibility because he says in the note that the injection was approved, but we end up having a bad feeling when we believe it is blepharitis. …” (N10).

Administering injections also brings greater responsibility, and the risk of doing something wrong can lead to insecurity. Patients not cooperating for various reasons was mentioned as challenging. One nurse expressed feeling insecure when having to take responsibility for a patient who could not fully cooperate:

“Yesterday we had a patient who claimed she never had received an injection in her left eye and today would be her first time … and this was kind of injection number 24 in that eye.... They (the patients) are growing old, some are a bit forgetful.” (N9).

Another nurse explained the danger of doing a patient harm:

“Of course I am happy when the patients get to keep their vision, but … it is no fun if you end up puncturing the whole eye, causing a retinal detachment, all because the patient could not lie still.” (N3).

### Theme 2: motivation

Traditionally, administering IVIs is a physician’s task. Mastering this task gave the nurses a sense of pride and a feeling of contributing to solving some of the departmentﹸs resource challenges. The nurses also valued being more involved in the treatment of patients.

#### Responsibility and respect

All of the 12 nurses agreed that the new task gave them increased respect from both patients and colleagues. The expanded repertoire of tasks also increased their responsibility, which the nurses felt sharpened them and made them better nurses. One nurse explained that she had to take better care of herself to be at her best:

“When I’m in the injection room, I’m more aware of my blood sugar. In the outpatient clinic, we measure visual acuity and eye pressure and I can feel my belly rumbling, but it’s okay, we keep going. But if I’m in the injection room, I’m more aware of that it affects me negatively and that I have to eat something before I continue.” (N8).

Another nurse said that nurses taking over new tasks and increased responsibility is the future:

“You feel the responsibility, but it’s a good kind of responsibility. This is the direction the world goes; we (the nurses) must do more and more ‘physician tasks’. It’s like this everywhere, with everything. It is a good development because we become more skilled professionally.” (N12).

The nurses appreciated learning a new task. One nurse emphasized that it was a privilege being certified to give injections:

“I get to be a part of something unique and special.” (N5).

Another nurse accentuated that learning a new procedure gave higher self-esteem:

“I feel that my skills have expanded. I learned a new procedure and mastered a new situation.” (N7).

#### To make a difference

The nurses expressed they accomplished something good by contributing to raising patients’ quality of life, and saving the department resources came as a bonus. One nurse explained why she felt more important when she was certified to give injections:

“I feel I do a better job when I am in the injection clinic than when I am doing other tasks. I feel like I make a difference when I administer injections.” (N5).

Another nurse highlighted the importance of helping patients have a better quality of life:

“I think it is exciting when I hear good news about the patient’s vision, because some patients actually get better visual acuity, and I think this is great … or at least they keep their visual acuity. It is fantastic to hear that they have better vision or that they stopped seeing skewed lines. I think this is very rewarding.” (N4).

### Theme 3: cooperation and confidence

The nurses agreed that collaborating with fellow nurses as a team could be both rewarding and demanding. A stable nursing team would provide safety for patients as they would not have to meet a new physician at every appointment. The nurses also agreed that a well-designed team of nurses could do a better job than the physicians. The nurses expressed that they felt confident administering injections after they had gained some experience.

#### Working as a team

Some days were busier than others, with over 30 patients receiving treatment on the same day. On days like these, the nurses highlighted the importance of working with people that they had good chemistry with. One nurse explained:

“It’s mostly positive working as part of a team, but sometimes it is not. … It depends on your energy level that day and what colleagues you cooperate with.”(N10).

An advantage of teamwork was the opportunity to seek support if something went wrong. One nurse explained what she would do if she ran into problems:

“We are very good talking things through, we nurses. If some things are difficult, I discuss it with my colleagues.” (N7).

#### Confidence

Several of the nurses mentioned that a nurse team worked more efficiently than the physicians because they were more focused on the task. In the focus group interview, the group dynamic made this especially clear, as the nurses agreed that their skills were as good as the physicians’ when it came to injecting anti-VEGF intravitreally. One nurse put it concisely:

“The point is that we (the nurses) do it better than the physicians.” (N12).

Another nurse added with a smile:

“The physicians feel their role is more serious; they don’t go along with the joke and the good vibe in the room. We nurses can have fun with the patient, but for the physicians it’s just a serious procedure.” (N11).

### Theme 4: evaluation

All the nurses expressed they felt safe administering injections after they had gained some experience. They also appreciated that the training was voluntary and that they could spend the time they needed. They all reflected on how the training program and the injection clinic could be improved. The nursesﹸ feedback resulted in a shorter and more intensive training program.

#### Continual learning

The nurses were overall satisfied with the training program. Some wished for continual learning with frequent lectures on relevant topics, more training in filling out the outpatient clinic form, and regular controls of the injection technique. One nurse stated:

“I would love to get a refresher along the way; I think that would be useful. Some theoretical repetition of blepharitis, for instance, and the rules when the patients need to postpone their injections. But the injection itself I think I have had plenty of training in.” (N6).

The nurses also mentioned that it would have been satisfying to learn more about both the theory of ophthalmic diseases and the diagnostics, for reasons including two that came up repeatedly—to satisfy own curiosity and to be able to answer questions from patients:

“It’s really as easy as learning to handle the slit lamp properly. Understand what it is you see. It’s easier to explain it to the patient when you have seen it yourself. They ask a lot. How does it look? Why is it like that?” (N3).

The nurses reported that patients often asked about their diagnosis and prognosis. Not being able to provide the answer, but having to refer them to a physician who could, took away some of that pride the nurses felt running the clinic.

“It’s a bit discouraging when the patients ask a lot of questions that I cannot answer; all I can say is that they should ask again in three months at your next appointment with the physician.” (N5).

#### Adjustments

The nurses preferred another nurse as a supervisor rather than a physician.

“I believe it’s much better when a nurse is the one giving instructions. I feel they think more about everything. What does the nurse in training need to know, observe, and try, and what progression should the nurse have?” (N11).

The nurses had opinions on what would make the day run smoothly. Electronic patient journals with missing information and too many patients on the injection list could cause stress. When they had to clarify information with a physician, this was time-consuming, as explained by one nurse:

“The physician will talk to the patient and time flies, and I have already prepared the patient and I am standing there waiting with the syringe in my hand. Several times I think that I must give the patient anesthetic eyedrops all over again.” (N10).

Giving one dedicated physician responsibility for answering questions was something all the nurses wanted. One nurse preferred shorter time between patients leading to more efficiency, but all the others favored the opposite. One of the nurses who wished for more time said:

“I want to talk more with the patients.” (N5).

## Discussion

Our study showed that the nurses trained to administer IVIs overall were satisfied with the training and reported that learning a new task led to higher self-esteem and increased respect. The nurses felt confident and in control when administering injections, although they experienced moments of insecurity. They had several suggestions on how to improve the training.

The training program was still under development while the first nurses were trained, and they could decide the progression of the training themselves. It became clear that a vertical task shift required changes in role identity and mindset [24]. As the first group of nurses embraced the new task, the nurses to follow likely adopted the new role identity and the new way of thinking, making training less time-consuming.

Good collaboration with colleagues is important because it makes the workday easier. Traditionally, physicians and nurses handle new challenges differently. While medical education highlights independence, responsibility, and confidence to rely on oneself, nursing education is more focused on care, communication, and cooperation [25]. If something proved difficult, the nurses handled this by discussing the problem with a fellow nurse. This team-oriented culture can encourage the nurses to take on untraditional responsibilities and increase the chances of a successful task shift [24]. The nurses experienced that they had to rely on teamwork to a greater extent in the injection room, which led to a new way of cooperating with fellow nurses, in contrast to the more traditional physician–nurse team.

The nurses mentioned several factors that motivated them to complete the training, including having a more varied workweek and making a difference in patients’ lives. These factors have previously been reported to influence the motivation to learn [26–28]. Another motivator was the pride and respect the nurses felt in mastering a new task. Taking responsibility for running the injection clinic may have initiated a desire in the nurses to learn more about ophthalmic diseases. Having to disappoint patients who have questions and refer them to a physician for answers may have taken away some of that pride. A study concluded that patients were less satisfied with the information provided by nurses about disease and prognosis [29].

The nurses mentioned eyelid infections as a source of insecurity and a common reason to turn to a physician for advice. Shifting tasks could lead to diffuse limits of liability [5]. Who will have the legal liability if malpractice occurs? This question is one concern considering the ethics and legislation around task shifting [30]. It may therefore be important to establish pre-defined limits of liability prior to a task shift.

The nurses in our study gained self-esteem and believed the way they administered the injections kept the patients calm and comfortable. Patient satisfaction has been recognized as an important factor for quality of care [31, 32]. The literature has shown that patients are satisfied with nurses delivering IVIs [33]. It is conceivable that shifting the administration of the injections to nurses ensures better continuity and that this makes the patients feel more satisfied [34, 35].

The suggested alterations to the training program that emerged during the interviews gave the department an opportunity to improve and adjust the training and the injection clinic [36]. As a result, a specific physician was designated to answer questions from the nurses. As more nurses were trained, it became clear that 10 days of intensive training was sufficient. At their own request, the nurses have a re-certification once a year to ensure quality and adherence to the procedures.

### Methodological considerations

This study adhered to the COREQ guidelines, which ensured transparency and trustworthiness of the findings and the interpretation of the data [23]. Our study included both individual interviews and a group interview. The safety and confidentiality of an individual interview differs from the dynamics in a focus group interview, where a common agreement will be highlighted [18, 19]. We experienced that the group dynamics brought broader perspectives and revealed new aspects of the training and the new task. The combination of interview methods strengthened the study, and the combination of different analysing tools visualized the data from a range of perspectives [21]. Further, a total sampling strategy was used, and none of the participants declined.

Conducting interviews came from a desire to learn from the trained nurses because an interview can give in-depth information on participants’ attitudes, thoughts, and actions [37]. This qualitative study is an important supplement to our previous RCT [10]. This mixing of methods can act complementarily and provide a richer and deeper understanding of the task shift concept [38], and it is in line with recommendations for training needs assessment [36].

Recruiting only highly motivated nurses who volunteered may have introduced a selection bias in that the most motivated nurses learn faster and may evaluate the training program in a more positive way [39]. However, utilizing dedicated and motivated nurses was most likely a criterion for success.

The first author had limited experience with the interview technique, and being inexperienced can make it more challenging to avoid being influenced by one’s own experience in interpreting the data [18]. However, the last author monitored the focus group interview and worked closely with the first author in interpreting the transcripts. Working at the department, the participants might have had a personal interest in the injection clinic becoming a success, which could have biased the feedback. On the other hand, the nurses seemed very communicative and gave both positive and negative feedback. The interviews were relatively short. However, the interviewer was familiar with the context, setting and participants, which ensured prolonged engagement, an important criterion for rigor in qualitative research [40].

## Conclusions

The nurses certified to inject anti-VEGF intravitreally expressed satisfaction with the training and the new task. Suggestions to improve the training were identified, which should be considered before it is implemented in other departments.

## Data Availability

The dataset analysed during the current study is available from the corresponding author on reasonable request.

## Abbreviations

Anti-VEGF: anti-vascular endothelial growth factor
DA: Dordi Austeng, second author
IVI: intravitreal injections
KHG: Kari Hanne Gjeilo, last author
SB: Stine Bolme, first author
RCT: randomized controlled trial
